# Peak detection in intracranial pressure signal waveforms: a comparative study

**DOI:** 10.1186/s12938-024-01245-9

**Published:** 2024-06-24

**Authors:** Miaomiao Wei, Solventa Krakauskaite, Sreya Subramanian, Fabien Scalzo

**Affiliations:** 1 Department of Electronic and Information, Zhongyuan University of Technology, Zhengzhou, China; 2 Department of Neurology, University of California, Los Angeles (UCLA), Los Angeles, USA; 3 Keck Data Science Institute, Pepperdine University, Malibu, USA

## Abstract

**Background:**

The monitoring and analysis of quasi-periodic biological signals such as electrocardiography (ECG), intracranial pressure (ICP), and cerebral blood flow velocity (CBFV) waveforms plays an important role in the early detection of adverse patient events and contributes to improved care management in the intensive care unit (ICU). This work quantitatively evaluates existing computational frameworks for automatically extracting peaks within ICP waveforms.

**Methods:**

Peak detection techniques based on state-of-the-art machine learning models were evaluated in terms of robustness to varying noise levels. The evaluation was performed on a dataset of ICP signals assembled from 700 h of monitoring from 64 neurosurgical patients. The groundtruth of the peak locations was established manually on a subset of 13, 611 pulses. Additional evaluation was performed using a simulated dataset of ICP with controlled temporal dynamics and noise.

**Results:**

The quantitative analysis of peak detection algorithms applied to individual waveforms indicates that most techniques provide acceptable accuracy with a mean absolute error (MAE) $\le 10$ ms without noise. In the presence of a higher noise level, however, only kernel spectral regression and random forest remain below that error threshold while the performance of other techniques deteriorates. Our experiments also demonstrated that tracking methods such as Bayesian inference and long short-term memory (LSTM) can be applied continuously and provide additional robustness in situations where single pulse analysis methods fail, such as missing data.

**Conclusion:**

While machine learning-based peak detection methods require manually labeled data for training, these models outperform conventional signal processing ones based on handcrafted rules and should be considered for peak detection in modern frameworks. In particular, peak tracking methods that incorporate temporal information between successive periods of the signals have demonstrated in our experiments to provide more robustness to noise and temporary artifacts that commonly arise as part of the monitoring setup in the clinical setting.

## Introduction

**Fig. 1:**
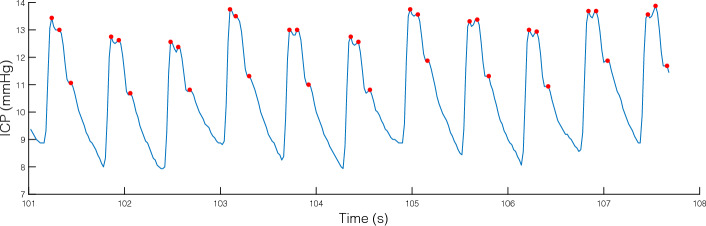
Example of continuous real-time monitoring of ICP waveform from a patient included in our dataset. Red dots depict the three peaks within each triphasic waveform

The monitoring of quasi-periodic biological signals such as arterial blood pressure (ABP), intracranial pressure (ICP), and electrocardiography (ECG) plays a fundamental role in the study of numerous disorders and diseases. These biological signals have something in common; they all exhibit characteristic variations that clinicians can use as markers of physiological change and provide additional insights through further analyses. This comparative study focuses on the ICP waveform, which is a quasi-periodic signal. As visualized in Fig. 1, each pulse can be associated with three peaks due to its triphasic nature [1]. Therefore, ICP morphological analysis often relies on identifying these three peaks. Based on their latency (i.e., time) and elevation (i.e., height), it is possible to characterize the morphology over time and then compute statistics of the ICP waveform for a particular time interval. We provide in this paper a comparative analysis of techniques for detecting the three peaks across the periods of the signal.

The study of the variations of the ICP signal is particularly important for patients of the NICU treated for traumatic injury (TBI) as the morphological variations observed in the ICP waveform may reveal the compensatory ability of the brain in the presence of cerebrovascular disruptions. Several computational frameworks have been developed to study how the changes observed in the morphology of the ICP signal are associated with the development of cerebral vasospasm [2], intracranial hypertension [3, 4], and abrupt changes in the cerebral blood carbon dioxide (CO2) levels [1, 5], and changes in the craniospinal compliance [6]. In addition to the average change of the ICP, studies [7, 8] have linked the morphology of the ICP waveforms with the prognosis of patients with a head injury. Hence, exploring ICP morphological characteristics such as peaks may help monitor pathophysiological intracranial changes.

Traditionally, peak detection in biological signals has been achieved using signal processing methods, including threshold-based and filtering methods [9, 10]. More recently, machine learning (ML)-based methods [11, 12] have been developed to solve this problem. ML methods are usually built on top of signal processing methods to obtain more robustness to noise by capturing the characteristics of the peaks and adapting to the noise profile of the signal specific to the context in which it is acquired. Many machine learning models are available, including neural networks, random forests, support vector machines (SVM), long short-term memory (LSTM) [13], etc. In the context of ICP analysis, these models can be grouped into two main categories depending on whether they are processing a single pulse at a time or if they are processing the continuous signal instead. It remains unclear, however, which methods are the best-performing ones on ICP signals.

This study compares ICP peak detection methods. After a technical review and description of the literature, we describe our peak detection experiments performed on actual and simulated ICP datasets.

## State-of-the-art

### Summary

Peak detection techniques on quasi-periodic signals can be grouped into two categories depending on whether they are processing individual waveforms or utilizing temporal patterns from previous waveforms to identify peaks in the current waveform. Traditionally, peak detection on individual waveforms has been achieved using signal processing techniques. In that scenario, a single beat/period of the signal waveform is used as input to a model, and the output corresponds to the latency and/or elevation of the peaks within that beat. It is common to assume that a peak is simply a local extrema of the curvature of the signal. In the context of peak detection in biomedical signals such as ICP, however, artifacts and noise make peak recognition challenging when relying on predefined heuristics only and often result in false positive detections. To tackle this problem, peak detection techniques have been improved using data-driven approaches (i.e., machine learning) that utilize a training set of data samples to learn a peak detection model. Data-driven techniques have demonstrated significant promise to improve the robustness of peak detection in biomedical signals.

Because strong correlations may exist between the peak locations of successive waveforms, tracking techniques such as Kalman filter, long short-term memory (LSTM), Bayesian inference, or MOCAIP (Morphological Clustering and Analysis of ICP Pulse) [14] can capture the temporal information between successive pulses to refine the location of the peaks. We review in the following sub-sections the techniques available in the literature to perform peak detection on individual (Section II-B) and continuous (Section II-C) waveforms.

### Peak detection on individual waveforms

Peak detection is assumed here to be performed on individual ICP pulses previously segmented from continuous ICP waveforms [10, 15]. In particular, we divide the single waveform methods into two sub-groups depending on whether they are based on signal processing only or if they also utilize data-driven models during processing.

#### Signal-processing techniques

In signal processing, it is common to think of peak detection as a search for a local extrema in the curvature of the signal. Most of signal-based methods utilize the local structure of the signal to identify the peaks. Among them, we identify threshold-based processes [16, 17], derivative-based techniques [18], and transform domain techniques [19–21]. Other methods perform peak detection by incorporating a larger context to describe the signature of each peak within the beat, which includes intensity weighted variance [22], filter-based techniques [23, 24], histogram-based techniques [25, 26], techniques using entropy [27], momentum [28], stochastic resonance [29], higher-order statistics [30], nonlinear energy operator [31], empirical mode decomposition [32]. More advanced techniques such as the wavelet transform and entropy of coefficients [33, 34] have also achieved promising results.

While peak detection techniques based on signal processing perform well on a wide range of applications, they encounter significant challenges when applied to real-world ICP data due to the variability across subjects, motion artifacts, and hardware acquisition noise characteristic of ICP waveforms. As described in the next subsection, these challenges have pushed researchers to utilize more robust techniques for these variations.

#### Data-driven techniques

Data-driven techniques utilize training data samples to infer a model optimized for peak detection. While the learning algorithm algorithms can take many different forms, many approaches formalize peak detection as a regression analysis problem between the input ICP waveform and the location of each peak (as the target output). This section gives an overview of ML methods used to detect ICP peaks and will be evaluated in our experiments. These methods include spectral regression (SR) [35], neural networks (NN) [36], support vector machines (SVM) [37], and extremely randomized decision trees (Extra-Trees) [38].

**Spectral regression (SR).** The SR algorithm [35] is a nonlinear regression method incorporating graph-based analysis with regularized linear regression. Assuming a set of *N* input data samples $\{x_0, x_1, \ldots , x_{N-1}\}$ and their corresponding predicted output $\{\hat{y}_0, \hat{y}_1, \ldots , \hat{y}_{N-1}\}$, the objective is to learn a regression model that outputs similar predictions $\hat{y}_i$ for input samples $x_i$ near each other in a graph representation The regression model is obtained by minimizing the following measure $\phi$:

1 $$\begin{aligned} \phi = \sum _{i,j=1}^N (\hat{y}_i - \hat{y}_j)^2\; W_{i,j}, \end{aligned}$$

where $W_{i,j}$ is the affinity matrix $W \in \mathbb {R}^{N \times N}$ that assigns a value to $W_{i,j}$ to indicate the similarity of the two input samples $x_i, x_j$; where *i*, *j* are used to represent the index of the $i^{th}$ and $j^{th}$ data samples, respectively.

While SR has been developed to solve linear problems, it can be extended to nonlinear problems using a kernel projection, which projects the original observation $x_i$ into a higher dimension using a nonlinear kernel. In the kernel version of SR, referred to as kernel spectral regression (KSR), the data input samples $x_i$ are replaced by the projected vectors in Eq.(2). In this study, we use the radial basis function (RBF) kernel:

2 $$\begin{aligned} R(x_i,x) = {\textrm{exp}}{(-\beta ||x_i-x||^2)},\; \beta > 0. \end{aligned}$$

**Neural networks (NN).** Neural networks is another popular machine learning model that can infer peak locations from an ICP waveform. Numerous neural network architectures exist; we focus here on a feed-forward network that comprises input, hidden, and output layers. The Levenberg–Marquardt algorithm [39] was used for its efficiency in training moderate-sized NNs. The SSE is used as the fitness function.

**Supper vector machine (SVM).** A support vector machine (SVM) [37] is a supervised learning method that constructs a set of hyperplanes in a high-dimensional space. SVM has been proven to be an effective tool in real-value function estimation. In the context of regression, SVM (also called support vector regression (SVR)) uses a n-dimensional tube to fit the data. During learning, the optimization process adopts an $\epsilon$-insensitive loss function, penalizing predictions farther than the threshold $\epsilon$ from the desired output. The value of $\epsilon$ determines the diameter of the tube; a smaller value indicates a lower tolerance for error and affects the smoothness of the overall predictions. For regression problems, SVM aims to identify the parameters of a set of hyperplane(s)/tube(s) that best fit the data using the following metric:

3 $$\begin{aligned} \sum _{s\in SV}^n (\alpha _s^+ - \alpha _s^-) R(x_s,x) + b, \end{aligned}$$

where *SV* is a subset of the input data samples *x* called “support vectors”, $\alpha ^+$, $\alpha ^-$ represents the learned dual coefficients, $R(x_s, x)$ is the response of the RBF kernel (Eq. 2) of the data sample $x_s$, and *b* is the bias.

**Extremely randomized decision trees (extra-trees).** Extra-Trees [38] is a regression method based on an ensemble of randomized decision trees. The learning of a randomized decision tree is performed by starting at the tree’s root node and successively splitting its left and right sub-trees. Each split (i.e., threshold) is obtained by sampling according to a Gaussian distribution estimated from the training samples. The process is repeated until a node has constant output values for all the training inputs. By building many randomized decision trees (e.g., $N>100$), the model can make predictions by using a new input through each tree and computing the average prediction across all the trees.

### Peak tracking on continuous waveforms

Although peak detection on individual pulses can achieve reasonable accuracy by identifying the signal signature of these peaks or learning a regression model between the waveform and the peak location with machine learning, processing pulses individually has some limitations. Hardware noise and human disturbance (such as motion artifacts) are inevitable in a clinical environment. These may cause distortion or even temporary loss of ICP waveform, making detecting and tracking peaks based solely on a single pulse challenging. Achieving continuous and real-time analysis of ICP waveforms is a high-level requirement of ICP monitoring in the NICU. Here, we describe techniques developed to process the continuous ICP signal and locate the peaks using temporal properties between successive beats as prior information, effectively tracking them across different periods. In the following, we describe Kalman filtering [40], Bayesian tracking [41], and LSTM (long short-term memory) [13], and MOCAIP (Morphological Clustering and Analysis of ICP Pulse) [14].

**Kalman filtering.** The Kalman filter algorithm [42] is a recursive algorithm that estimates the distribution of unknown variables from the measured noisy data. After several iterations, the estimated value is expected to converge to the actual value of the unknown variables; the location of the peaks in our case. The process is efficient as it only needs the current measured input, the previous state, and the uncertainty state matrix to calculate the predicted value when the subsequent measurement is observed. The Kalman filter is composed of a prediction and an updating step.

The state variable $\hat{x}_k^{-}$ and its covariance $P_k$ are estimated during the prediction step:

4 $$\begin{aligned} \hat{x}_k^{-}=\, {} A \hat{x}_{k-1}+B u_k, \end{aligned}$$

5 $$\begin{aligned} P_k^{-}=\, & {} AP_{k-1}A^T+Q, \end{aligned}$$

where *A* is the state-transition matrix, *B* is the control-input model, and *Q* represents the covariance of the noise.

During the updating step, these estimates are evaluated using a weighted average, such that a greater weight is set to estimations with greater confidence:

6 $$\begin{aligned} K_k=\, & {} {P_k^{-}H^T(HP_k^{-}H^T+R)+Q}^{-1}, \end{aligned}$$

7 $$\begin{aligned} \hat{x}_k=\, & {} \hat{x}_k^{-}+K_k(z_k-H\hat{x}_k^{-}), \end{aligned}$$

8 $$\begin{aligned} P_k=\, & {} (I-K_k H)P_k^{-}, \end{aligned}$$

where *H* represents the observation matrix, $\hat{x} _{k}^{-}$ and $\hat{x}_k$ are the prior and posterior state estimates at step *k*. *R* is the measurement error covariance, $z_k$ and $u_k$ are the measurements and the control vectors at step *k*, and *K* represents the Kalman filtering gain.

**Bayesian tracking.** Nonparametric belief propagation (NBP) [43] is a probabilistic inference algorithm applied in computer vision to track the movements of people, animals, robots, cars, etc. We previously used NBP [41] to track ICP peaks in real-time. Bayesian inference associates continuous probability distributions as the location of each peak.

During detection, NBP utilizes a dynamic graph where nodes represent the location of each peak. Information between the different peaks of a current pulse, and between the peaks at the prior time point are propagated in the graph via a message-passing algorithm called Belief propagation. At the *n*th iteration, the message *m* passed from node *a* to *b* is expressed as:

9 $$\begin{aligned} m_{a,b}^n(h_a)\leftarrow \int \phi _{a,b}(h_a,h_b)\phi _a(h_a,o_a) \prod _{c\in C_{a\backslash b}} m_{c,a}^{n-1}(h_a) dh_a, \end{aligned}$$

where $h_a\in \textbf{h}$ represents the hidden variable at node *a*. $C_{a\backslash b}$ represents the set of nodes connected to *a* (except node *b*). $\phi _{a}(h_a,o_a)$ is the observation potential between hidden variable $h_a$ and observation variable $o_a$ of node *a*, $\phi _{a,b}(h_a,h_b)$ is the compatibility potential between hidden variables $h_a$ and $h_b$. After several iterations, the approximation of $n^{th}$ iteration $\hat{p}^n(h_a|o)$ converges to the true marginal distribution $p(h_a|o)$ is:

10 $$\begin{aligned} p(h_a|o)\sim \hat{p}^n(h_a|o)\leftarrow \phi _a(h_a,o_a)\prod _{b\in C_a} m_{a,b}^n(h_a). \end{aligned}$$

In NBP, the message $m_{a,b}(h_a)$ is expressed as a mixture of *D* kernels:

11 $$\begin{aligned} m_{a,b}(h_a)= \frac{1}{D}\sum _{i=1}^D\omega _a^i N(h_a;\mu _a^i,\Sigma _a^i), \end{aligned}$$

where $\omega _a^i$ is the weight of the *ith* kernel with mean $\mu _a^i$ and variance $\Sigma _a^i$. *D* is the number of particles used for estimation. The observation potential is represented as weighted mixtures of Gaussian density functions.

**Long short time memory (LSTM).** LSTM [13] is a type of recurrent neural network (RNN) that allows information to persist inside the network via loops in its architecture. LSTMs are particularly well suited to represent time series such as ICP waveforms. An LSTM cell is defined by a state that changes according to three types of gates:

- Input gates $\mathcal {I}_t \in \mathcal {R}^N$ update the state of the cell and decide which values should be updated.
- Forget gates $\mathcal {F}_t \in \mathcal {R}^N$ are used to select relevant information with respect to a previous state.
- Output gates $\mathcal {O}_t \in \mathcal {R}^N$ determine the final cell state and the output value.

Given an input sequence $x = \{x_1, x_2, \ldots , x_T\}$ of length *T* with corresponding memory cell unit $C_t \in \mathcal {R}^N$ and hidden unit $h_t \in \mathcal {R}^N$ at time *t*, the parameters of the model are updated sequentially, as follows:

12 $$\begin{aligned} \mathcal {F}_t=\, & {} \sigma (W_{f}.[h_{t-1},x_t] + b_f), \end{aligned}$$

13 $$\begin{aligned} \mathcal {I}_t=\, & {} \sigma (W_{i}.[h_{t-1},x_t] + b_i), \end{aligned}$$

14 $$\begin{aligned} \mathcal {O}_t=\, & {} \sigma (W_o[h_{t-1},x_t] + b_o), \end{aligned}$$

15 $$\begin{aligned} \tilde{C_t}=\, & {} \tanh (W_c.[h_{t-1},x_t] + b_C),\end{aligned}$$

16 $$\begin{aligned} C_t=\, & {} \mathcal {f}_t * C_{t-1} + \mathcal {I}_t * \tilde{C_t}, \end{aligned}$$

17 $$\begin{aligned} h_t=\, & {} \mathcal {O}_t * \tanh {C_t}. \end{aligned}$$

The function $\sigma (x) = 1/(1+e^{-x})$ used to compute $\mathcal {F}_t, \mathcal {I}_t, \mathcal {O}_t$ is a sigmoid function whose values lie within the range [0, 1]. In addition to input, forget, and output gates previously described, the LSTM makes use of a memory cell unit $C_t$ obtained from the sum of the previous memory cell unit $C_{t-1}$ modulated by $\mathcal {F}_t$, and a function of the current input $x_t$ and previous hidden state $h_{t-1}$ modulated by the input gate $\mathcal {i}_t$. The output gate $\mathcal {O}_t$ is then used to determine what parts should be considered and then multiplied with the $\tanh$ of the memory cell state $C_t$ to produce the hidden unit $h_t$. By learning how much of the memory cell state $C_t$ should be transferred to the hidden state $h_t$ based on the input $x_t$ and previous state, this structure allows the LSTM to capture complex temporal dynamics such as the ones present across ICP waveforms.

**MOCAIP algorithm.** The Morphological Clustering and Analysis of ICP Pulse (MOCAIP) [14] framework was designed to extract morphological variations of ICP pulses. MOCAIP utilizes a Gaussian distribution as prior model for the peak location. The detection of ICP peaks is performed through the three main following steps:

- Pulse segmentation: The continuous ICP signal is segmented into a series of individual pulses using a dedicated algorithm [44] that utilizes ECG QRS markers [45]. A hierarchical clustering algorithm is utilized to extract a representative pulse over a segment of 1 min.
- Peak candidates detection. Candidate peaks are detected on the ICP pulse using its second derivative. They are extracted from the convex region and the concave region on the ascending edge of the signal or the concave part and the convex part on the falling edge of the signal.
- Peak Designation. The three peaks are selected from the set of candidate peaks such that they maximize the likelihood of belonging to a previously trained Gaussian mixture model (i.e., prior model).

## Methods

### Problem formulation

**Fig. 2:**
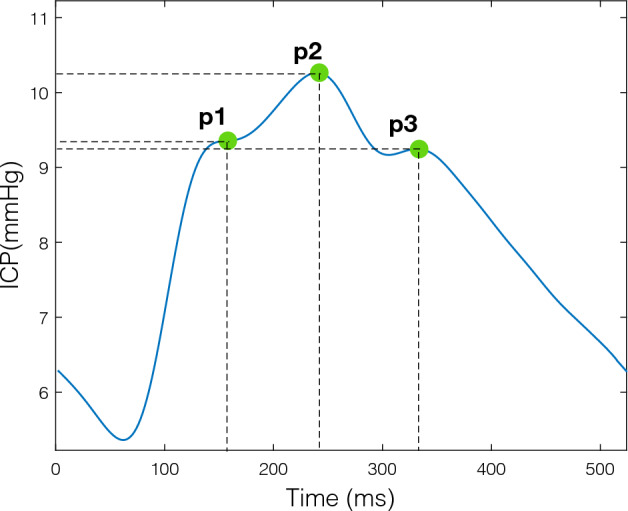
Illustration of a segmented pulse from a continuous ICP waveform and its three peaks (p1, p2, p3)

When acquired at a high enough frequency, ICP signals typically exhibit a sequence of waveform pulses such that each pulse includes three peaks, as illustrated in Fig. 2. We decompose the peak detection process on a raw signal by assuming that the continuous ICP waveform has been segmented into a set of individual beats ($s_1, s_2, \ldots , s_n$) using a standard beat segmentation algorithm [10, 15]. This is generally achieved with high accuracy - especially when the ECG signal is available. Assuming a segmented ICP waveform, we focus on two formulations of the peak detection problem. In the first case, we consider the task of detecting the three peaks within a single ICP pulse. In the second case, the peak detection is achieved by a tracking algorithm that exploits the estimated position of the peaks from previous pulses. In both formulations, a peak location is defined in terms of its temporal location $l \in \mathcal {R}$ and intracranial pressure elevation $e \in \mathcal {R}$, such that $p_{i \in {1,2,3}} = \{l,e\}$ denotes the *i*th peak of the pulse. The goal is to obtain automatically the position of the peaks in each beat $s_i$ using a peak detection algorithm $P_d$, which can be denoted as $P_d(s_i) = \{p_1, p_2, p_3\}$.

### ICP data

#### Clinical dataset

The dataset of ICP signals used in this study was collected from 64 patients receiving treatment for various ICP-related disorders in the Neuro ICU. The ICP was acquired using intraparenchymal microsensors placed in the right frontal lobe. The raw ICP waveform was recorded continuously at a sample rate of either 240Hz or 400Hz. 153 segments of ICP signal lasting almost 5 h were extracted. ICP and ECG signal were then pre-processed to segment individual beats to produce a set of 14,230 raw pulses. Among them, 13,611 valid pulses were obtained and formed the clinical dataset used for our simulation. The dataset is particularly challenging because there is a large variability in the ICP signals due to each patient’s condition.

In our experiments, the raw ICP waveforms were pre-processed before being used as input for single waveform or tracking analysis. The learning models used require a fixed length for the input data. ICP waveforms were first resampled to a fixed length because the waveforms’ lengths are dependent on the patient’s heart rate, which is variable. Each beat sample $\vec {S_i} \in S$ was resampled to a vector of 400 values. A left shift was then performed to align the beats. We define the alignment point to be the minimum of each beat waveform:

18 $$\begin{aligned} \text {start} = \mathop {\mathrm {arg\,min}}\limits _i{(\vec {Z}_i)}, \end{aligned}$$

and perform a circular shift to set this point as the first element of each beat vector, where *n* is the length of $\vec {Z}_i$.

19 $$\begin{aligned} \vec {N}_i = \vec {Z}_{(i+{\text {start}}){\text {mod}}(n)}. \end{aligned}$$

Since there is usually noise in ICP waveforms, which results in the distortion of the waveform, especially for sharp noise, the absolute magnitude of the waveform can be unreliable in clinical settings. To reduce this impact, each sample $\vec {X}_{i}$ is normalized so that its AUC is 1.

20 $$\begin{aligned} \vec {X}_{i,j} = \frac{ \vec {X}_{i,j} }{ \sum _{j=1}^J \vec {X}_{i,j}} \end{aligned}$$

Three experienced researchers established the groundtruth by reviewing each ICP pulse and manually assigning the position of the three peaks. Specifically, the researcher’s task was to select the suitable peak candidates for each peak (p1, p2, and p3) among those automatically detected at curve inflections. Researchers cross-validated their results and, if necessary, harmonized them using the annotation of the previous and following pulses as reference. For a few difficult cases where the researchers could not agree on the position of some peaks, the pulse was removed from the dataset. This procedure ensured that the groundtruth is not biased to a specific researcher. A custom-made annotation tool allowed for flagging missing peaks. In our dataset, $p_1$ was missed in 1717 pulses, $p_2$ in 265 pulses, and $p_3$ in 34 pulses. Data from two patients were removed due to the device malfunction. The data were acquired at the Ronald Reagan Medical Center at the University of California, Los Angeles (UCLA), and the UCLA Internal Review Board (IRB) approved the usage of this archived dataset.

#### Simulated dataset

To verify the effectiveness of the peak detection algorithms under controlled variability, we created a simulated dataset of ICP waveforms. A probabilistic generative model was used to simulate realistic shape variations of an ICP pulse. The model was formalized as a Gaussian Mixture Model (GMM) composed of three Gaussian components. The Gaussian Mixture model is a linear combination of Gaussian distributions:

21 $$\begin{aligned} p(x) = \Sigma ^K_{k=1}\pi _k\; \mathcal {N}(x|\mu _k,\Sigma _k), \end{aligned}$$

where $\pi _k$ is the weight associated with the $k^{th}$ component, and the number of components *K* was set to 3 in our experiments. The parameters $\pi _k, \mu _k,\Sigma _k$ were fitted using the clinical data using a random sample of 1, 500 ICP waveforms.

**Fig. 3:**
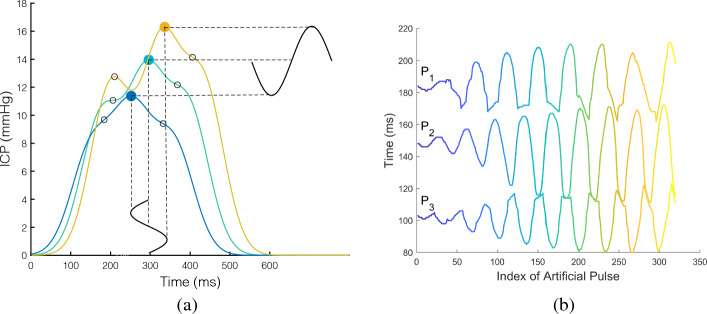
Illustration of simulated ICP waveforms from our GMM generative model (**a**) and corresponding latency for the 3 peaks (p1, p2, p3) over a sequence of generated data (**b**)

A series of pulses was then generated from this GMM model by incorporating an independent temporal change $c_{i\in {1,2,3}} = sin(z)$ on the mean of each component $\mu _k \in \mathcal {R}^2$. The model of the temporal dynamic was formalized as a sine wave function whose value $c_k$ was added to its corresponding mean $\mu _k$. It should be noted that two independent sine functions were used: one that acts on the latency and the other on the pressure of each peak. The generative model was then used to reconstruct individual waveform pulses at a sampling rate of 400Hz. The range of the sine wave was constrained by the fluctuation range observed in our clinical datasets. Figure 3 illustrates the variations induced by the sine wave on the latency of the three peaks.

### Experiments

Our experiments aim to compare the accuracy of several machine-learning models in locating the peaks within the ICP signal. For both the clinical and simulated datasets, we evaluate the accuracy of the models in detecting the peaks under a varying amount of noise. We also perform evaluations to evaluate the robustness to missing data.

#### Experiment #1: peak detection on clinical dataset

The evaluation performed as part of this experiment is carried out on individual waveforms where the input provided to the regression model does not include any context or waveforms from previous time points. The algorithms evaluated in our benchmark are spectral regression (SR), kernel spectral regression (KSR), neural networks (NN), support vector machines (SVM), and long short-term memory models (LSTM).

A tenfold cross-validation, performed at the patient level, is performed separately on the clinical and simulated datasets. For each training iteration, a threefold cross-validation is used on the training set to optimize the hyperparameters - this procedure is commonly referred to as a nested cross-validation. The ICP waveforms with missing peaks were included as part of the experiments. However, the missing peaks were ignored from the computation of the error. The input provided to the machine learning algorithms for both datasets is the ICP waveform re-sampled at 400Hz. The mean absolute error (MAE) and the root mean square error (RMSE) are used as a metric of accuracy and computed per peak and for each algorithm:

22 $$\begin{aligned} \text {MAE}=\, & {} \frac{|y_i-\hat{y_i}|}{n}, \end{aligned}$$

23 $$\begin{aligned} \text {RMSE}=\, & {} \sqrt{\frac{\sum _{i=1}^n(y_i-\hat{y_i})^2}{n}}, \end{aligned}$$

where $y_i$ represents the *ith* observation, $\hat{y_i}$ is the prediction of $y_i$ for the given model, and *n* denotes the total number of observations. The average error is computed across the 3 peaks between the actual value of the peaks $y_i = (p_1, p_2, p_3)$ and the prediction $\hat{y}_i = (\hat{p}_1, \hat{p}_2, \hat{p}_3)$ of the regression method.

The monitoring of ICP can be adversely impacted by various noise and artifacts (including electromagnetic interference from other equipment and self-noise). In practice, it is manifested by abnormal fluctuations in the ICP waveform. To reflect these signal perturbations and evaluate the robustness of peak detection algorithm to them, we create noisy replication of our ICP datasets by adding varying uniform random noise levels (from 5 to $15\%$ of the signal range) on the original ICP waveform.

Hyperparameters were optimized using nested cross-validation using only the training folds at each iteration. Specifically, we list below the optimized parameters for each method and list the implementation source. Matlab implementation of spectral regression and kernel spectral regression was obtained from Prof. Deng Cai’s academic website at http://www.cad.zju.edu.cn/home/dengcai/. The spectral regression hyperparameters were the kernel type used for the affinity matrix *W*, the regularizer parameter $\alpha$, and the number of neighbors used to compute *W*. For kernel spectral regression, an additional hyperparameter was used to control the standard deviation of the RBF kernel, which was also the optimized hyperparameter for SVM. For the neural network, the number of hidden layers/nodes, learning rate, optimizer were optimized. For the random forest, we optimized the number of decision trees. For LSTM, the number of hidden nodes was the only parameters fine-tuned. Except for spectral regression and kernel spectral regression (obtained from Deng Cai), the implementation of all the methods obtained from Matlab official toolboxes as of version R2022a.

#### Experiment #2: peak detection simulated dataset

In this second experiment, the evaluation is conducted on a series of ICP pulses. In particular, we assume that a regression algorithm first predicts the position of the 3 peaks. Such prediction, affected by noise and artifacts, is then filtered using a tracking algorithm to obtain a refined position of the peaks by utilizing temporal dependencies between successive ICP pulses. The tracking algorithms evaluated are MOCAIP [14], nonparametric Bayesian tracking [41], Kalman filter [42], and LSTM [13]. The regression model used to obtained candidate peaks on single waveforms is KSR. Similarly to our previous experiment, we repeat the evaluation by adding various noise levels (5–15%) to the simulated data. The noise was uniformly distributed relative to the range of the data and added independently to the latency and elevation values.

All the tracking code was implemented in Matlab and executed under the version R2022a. MOCAIP and the Bayesian version of MOCAIP are available on GitHub under https://github.com/NeuroResearchCore/trackLight.

In some cases, the patient’s movement or other physiological activities will cause a loss of connectivity, resulting in data loss in the ICP waveform. Without a signal, traditional ICP peak detection algorithms based on a single waveform will fail. We modified the simulated dataset to set some intervals to null to simulate this situation. This helps verify whether the tracking algorithm can utilize prior information to keep track of the peak over time. To simulate the missing ICP waveform, we divide the simulated waveform into several groups, and two or three missing segments of various lengths (2–4 pulses missing) are produced in each group to ensure the randomness of the missing situation and the dispersion of its distribution in the whole waveform.

## Results

### Experiment #1: peak detection on clinical dataset

The mean absolute error (MAE) of six peak detection algorithms on individual waveforms is reported in Table 1 after a tenfold cross-validation. The table summarizes the results for each of the three peaks. Each sub-table corresponds to the performance concerning one of the peaks. The fourth sub-table represents the average performance across all peaks. The columns correspond to the noise levels (0–15%). The MAE values (in milliseconds) were mapped to a color such that blue indicates lower error, and yellow indicates higher error.

On average, the results on the clinical dataset show that the error is the smallest for $p_2$, followed by $p_1$, and finally, $p_3$. This is because the position of $p_2$ is more stable than other peaks. Without added noise, KSR, SVM, and Random forests perform best (RMSE = 0.08, MAE $\le 4$ ms) when considering the average of the three peaks. In the presence of $15\%$ noise, the estimated error of KSR is the smallest (RMSE = 0.13, MAE $\le 10$ ms) as it appears to be less affected by noise. As expected, the MAE and RMSE of all algorithms increases due to the noise level. We note that not all algorithms grow at the same rate than the noise. For example, the error of spectral regression and neural networks increases much higher than in other methods.

Similarly, Table 2 provides the results after evaluating the peak detection methods on the simulated dataset. When the error is averaged over the three peaks, KSR offers the best performance among the six algorithms regardless of the noise level. The RMSE of spectral regression, LSTM, neural network, and random forests are higher (RMSE $\ge 0.29$, MAE $\le 10$ ms) and are greatly affected by noise. From the results summarized in Tables 1 and 2, we conclude that KSR performs better than the algorithms when considering a single waveform at a time.

**Table 1:**
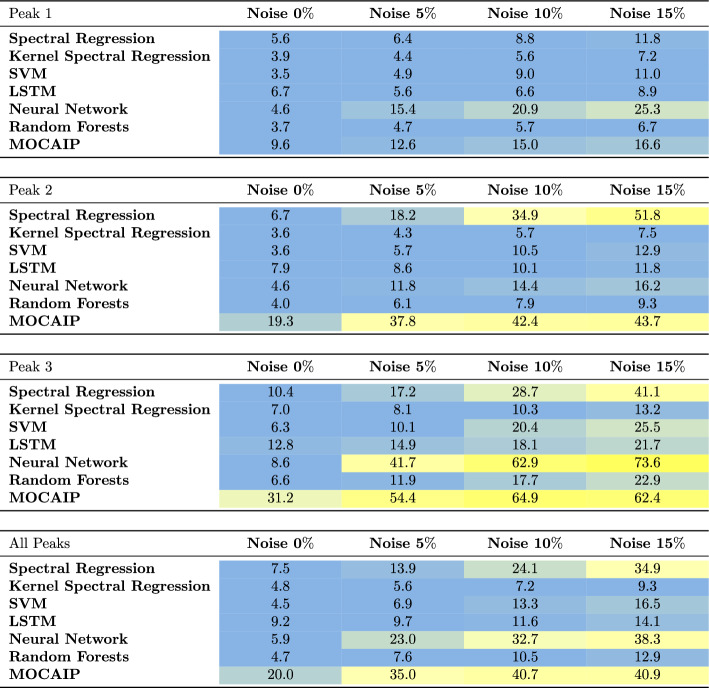
Performance of peak detection algorithms in terms of mean absolute error in milliseconds (ms) after evaluation on clinical ICP data with varying noise levels (from 0 to $15\%$)

**Table 2:**
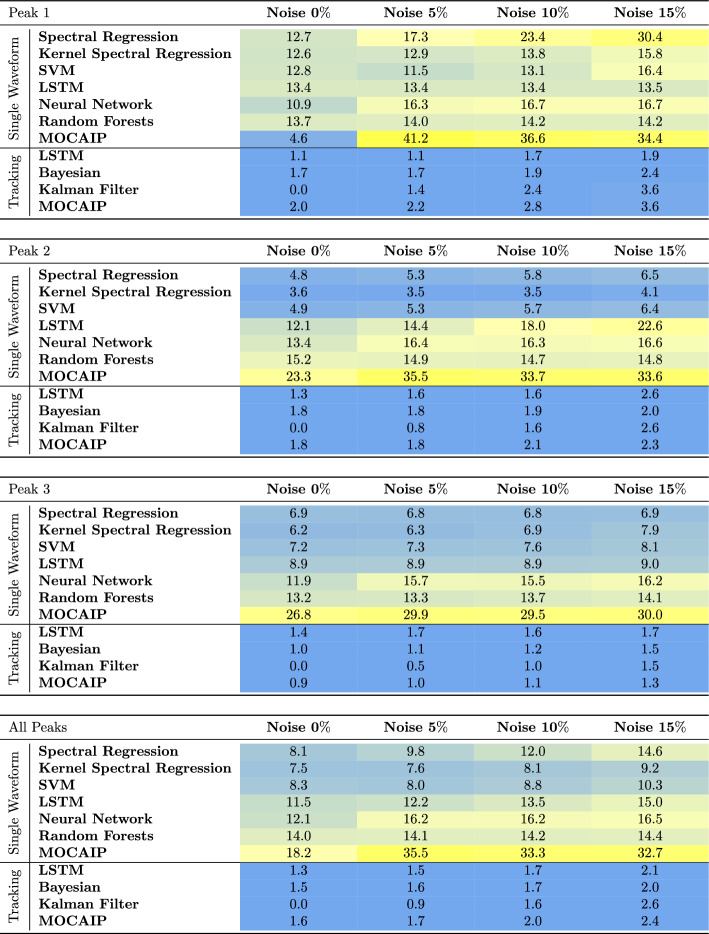
Performance of peak detection and tracking algorithms in terms of mean absolute error in milliseconds (ms) after evaluation on simulated ICP waveforms with varying noise levels (from 0 to $15\%$)

### Experiment #2: peak detection on simulated dataset

**Fig. 4:**
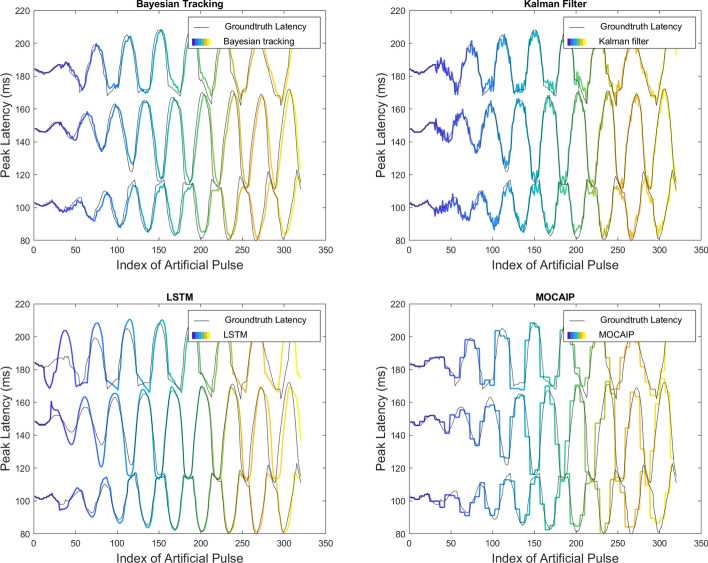
Tracking results on a simulated ICP dataset with 5$\%$ additive noise. Color curves represent the filtered peak latency on our simulated dataset using four different tracking models: Bayesian tracking, Kalman filter, LSTM, and MOCAIP

**Fig. 5:**
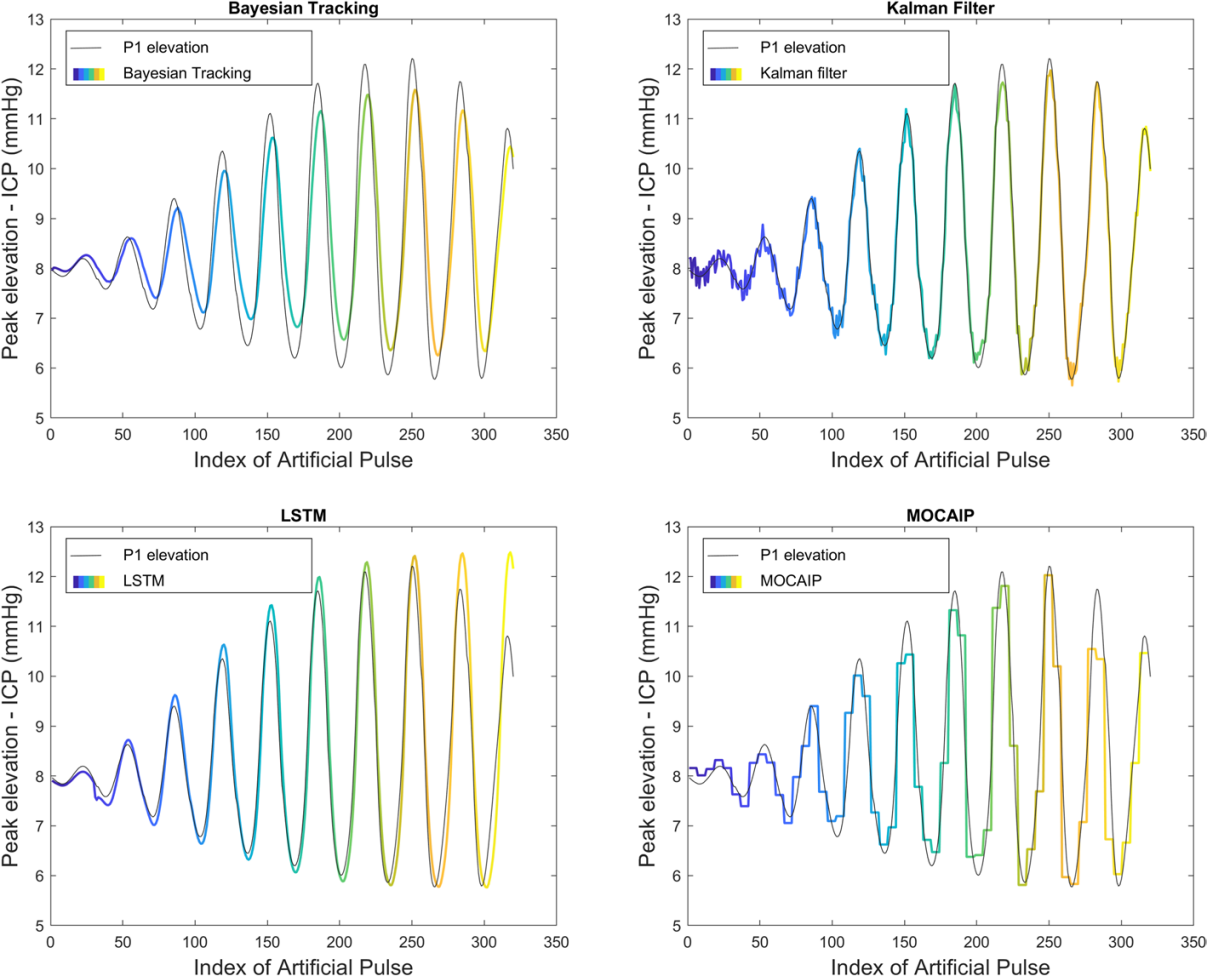
Tracking results on a simulated ICP dataset with 5$\%$ additive noise. Color curves represent the filtered elevation of the first peak on our simulated dataset using four different tracking models: Bayesian tracking, Kalman filter, LSTM, MOCAIP

The results of the peak tracking methods (Bayesian tracking, Kalman filter, LSTM, and MOCAIP) on continuous ICP are summarized in Table 2 and illustrated in Figs. 4 and 5. In Fig. 4, each plot includes the latency of the peak with noise (gray) and the filtered position of the tracking algorithm (color curves). These curves are repeated for each of the three peaks ($p_1$, $p_2$, and $p_3$), which can be judged according to the value range of its *Y*-axis. Figure 5 displays the results regarding the elevation of the first peak. For better visibility, we opted only to show the tracking of the first peak, as all peaks tend to be within the same elevation range in our simulated dataset.

The results illustrated in Table 2 indicate that all tracking methods outperform single waveform techniques, especially in high noise. All tracking techniques perform equally well with a RMSE of 0.04–0.05 and MAE $\le 3$ ms. This result is confirmed across the three peaks.

The accuracy of each tracking algorithm can be observed based on how close the estimate is to the groundtruth (shown in Fig. 3b). The tracking results of the Bayesian tracking and Kalman filtering framework on the three peaks closely follow the original peak latency. Although the signal is affected by noise, the tracking results still reflect the trend of the original position very well. We can conclude that the tracking algorithm effectively tracked the continuous waveform under this noise setting (i.e., $5\%$). The tracking result of LSTM is inconsistent with the groundtruth location, and its tracking result behaves differently for each peak and in different periods. The tracking result of $p_1$ is better than that of $p_2$ and $p_3$. For $p_3$, the detection is poor at the beginning and end of the tracking. The initialization phase of the LSTM could cause inconsistency in the initial part. Finally, the tracking result of MOCAIP captures the overall variations of the peak location but does not offer the same level of granularity as other techniques. MOCAIP is based on a clustering process to achieve peak detection. The input data are obtained by using a 1-min cluster average. However, it is worth mentioning that MOCAIP does not require a training process.

**Fig. 6:**
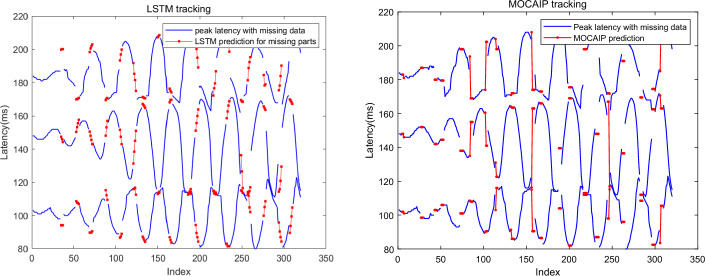
LSTM and MOCAIP tracking results of peak latency are illustrated in the presence of missing data segments. Blue segments represent the peak latency detected on observed ICP waveforms, while the red segments represent the peak latency estimated in the presence of missing ICP waveforms

The tracking results in the presence of missing data are illustrated in Fig. 6, where the blue curve represents the waveform with missing segments, and the red represents the inferred output using one of the tracking algorithms. To enhance contrast, only tracking results for the missing data are displayed. The missing data segments are randomly distributed in the whole waveform range. In most cases, the tracking algorithm recovers the missing data by effectively capturing the trend of the data.

By observing the output predictions of the MOCAIP algorithm, we can see it can approximate the trend of the peak position even when data is missing. Although MOCAIP does not follow the details of the changes, it is still useful for getting an approximate estimation for missing data segments. On the other hand, LSTM provides a more refined estimate of the missing data. It should also be pointed out that only LSTM and MOCAIP algorithms are used for missing data simulation because both Bayesian tracking and Kalman filter frameworks rely on the input for tracking, and the frameworks will not work when no input data are provided.

## Discussion

Over the last two decades, machine learning algorithms have produced significant breakthroughs in various domains. In this study, we demonstrated the ability of several machine learning models to achieve high accuracy in a peak detection problem on a quasi-periodic signal, the intracranial pressure signal (ICP). Among the evaluated techniques, the peak detection error of kernel spectral regression (KSR) was the lowest, whether based on simulated or clinically acquired data.

We provided comparative results regarding tracking methods used to filter the peaks continuously. Bayesian inference, Kalman filtering, LSTM, and MOCAIP algorithms can represent the temporal dependence of neighboring pulses in the peak prediction process. The results of our experiments show that such frameworks are remarkably robust to noise and missing data. This could be explained by the fact that the temporal dependencies can play a significant role in maintaining the correct position of the peaks over time, as they are unlikely to change drastically between successive heartbeats.

ICP pulses arise from the blood pressure variation in the cerebral vasculature. In an ICP pulse, the specific distribution of sub-peaks is affected by capillary, arterial, and venous blood pressure pulses and their interactions with three major intracranial parts, including the brain tissue, the cerebral vasculature, and the cerebrospinal fluid circulatory system. Consequently, it is conceivable that ICP pulse morphological changes may provide reasonable indications of changes in these compartments. Also, these changes can be triggered by various pathological incidents, such as the narrowing cerebral arteries (vasospasm) after subarachnoid hemorrhage and the development of mass-occupying lesions after a brain injury. Therefore, the long-term continuous monitoring and recording of the ICP waveform provide the changing trend of the patient’s physical condition, which is helpful for doctors to conduct pathological analysis of the state. Moreover, the tracking algorithm can predict the position of the ICP peak in a short period, which is also helpful for predicting the development of the disease in the clinical setting. In addition, given the interaction between biological signals, further study on the relationship between ICP and other biological signals to assist ICP waveform analysis is another direction to improve peak detection technology.

While we have made a special effort to identify techniques relevant to peak detection in ICP signals, the list of methods we have compared is not meant to be exhaustive. However, the set of experiments and the data can provide a baseline accuracy for developing and benchmarking future peak detection and tracking methods on ICP.

All the data and code used as part of our experiment will be made publicly available on the lab website of Prof. Scalzo (http://www.fabiens.net). To the best our knowledge, this would become the first publicly available and curated dataset of ICP signals with both simulated and clinical sources. This is provided with the hope that the data and experimental protocol can serve a as benchmark for the development and evaluation of future peak detection methods in ICP.

## Conclusion

This paper demonstrates that tracking of ICP waveform morphology can be performed in real-time with high accuracy using machine learning models such as kernel spectral regression (KSR), support vector machine (SVM), and LSTM. The acquisition of the ICP signal in a neuro-intensive care unit is often associated with signal loss and severe artifacts. To address these issues, our study demonstrated that peak detection models can be coupled with tracking models such as Kalman filter and nonparametric Bayesian inference to obtain robustness to temporary signal loss and improve the detection accuracy of the three landmarks. This paper also provides an ideal framework to benchmark future peak detection and tracking models. Although these tracking frameworks are demonstrated on ICP waveforms, they could, in principle, be used as part of the detection process of other quasi-periodic biological signals, such as ECG and CBFV.

## Data Availability

Data and code to replicate experiments will be available on Prof. Fabien Scalzo’s website http://www.fabiens.net.
